# The Pathology of Suppuration in the Nasal Accessory Sinuses and Its Relationship to Nasal Polypus

**Published:** 1899-07-29

**Authors:** Charles H. McIlraith

**Affiliations:** Senior Clinical Assistant (late Resident Medical Officer), the Throat Hospital, Golden Square, W.; late Aural Assistant, the London Hospital


### Hospital Clinics and Medical Progress.

### THE PATHOLOGY OF SUPPURATION IN THE NASAL ACCESSORY SINUSES AND ITS RELATIONSHIP TO NASAL POLYPUS.

By Charles H. McIlraith, M.A., M.D., &c. (Glasg.), Senior Clinical Assistant (late Resident Medical Officer), the Throat Hospital, Golden Square, W.; late Aural Assistant, the London Hospital.

In studying the pathology of the morbid condition or conditions on which suppuration in the nasal accessory sinuses depends, we must consider what takes place in inflammatory conditions of the nasal mucous membrane, seeing that the mucous membrane lining the cavities is continuous, and almost identical in structure, with that in the nose.

Inflammation occurring in the nose begins with an hyperæmia of the mucous membrane. This becomes swollen, and is frequently studded over with minute patches of ecchymosis, due to rupture of some of the fine blood-vessels ramifying in it. At first this swelling may be so great as to partially obstruct the nasal passages, and so give rise to the feeling of having "a stuffed nose." In this stage there is little or no exudation. As a rule there is a sensation of abnormal dryness, the secretions from the tumified mucous membrane and the glands opening on it being diminished. Later on the swelling increases, being then largely due to œdematous infiltration, which involves not only the superficial but the deeper layers of the mucous membrane, including the periosteum. This exudation, consisting of blood plasma, and leucocytes, soon finds its way to the surface, and is poured out as a profuse, thin, watery, and somewhat acrid discharge of sero-mucus, with probably some blood-cells. Then the swelling subsides somewhat, the discharge becoming more serous in character, and after a time, possibly some weeks, leucocytes are poured out more abundantly, the secretion gets thick, opaque, and yellowish or greenish, and there is finally a discharge with all the characters of muco-pus or pus.

As a rule, the acute inflammatory process soon results in resolution, the tumefaction and secretion gradually subsiding and the mucous membrane returning to its healthy state. Occasionally, however, and especially after one or two recurrent attacks, resolution is more and more slow, and a chronic morbid process results; one of the most frequent results of which is the establishment of what may be called a myxomatous condition of the mucous membrane. In this the tissue, which still presents the ordinary characters of inflamed mucous membrane, viz., a meshwork of connective tissue with large serous spaces, with here and there dilated mucous glands scattered through it, with round-celled infiltration depending on the extent and acute character of the inflammatory process and covered superficially with ciliated epithelium, becomes so infiltrated with serous fluid as to develop a soft, jelly-like thickening of the tissue. In some cases this goes on to the formation of mucous polypi, which grow, sometimes single, sometimes in quantity, and often to large size, completely blocking up the nasal passages.

That nasal polypi are generally altered hypertrophic conditions of the nasal mucous membrane is, I think, the view now held by most rhino-pathologists. While admitting, however, that hypertrophic rhinitis and polypi are allied conditions, that the one is the outcome of the other I cannot believe. A hypertrophic and a myxomatous condition of the nasal mucous membrane are by no means the same. While in the former condition there is always an excess of connective tissue with blood spaces amongst it, in the latter, even from the first, the connective tissue becomes more and more scanty, and the blood spaces are replaced by spaces filled with mucus or sero-mucus. In numbers of cases, also, we meet with polypi growing with slender pedicles in which there is no true hypertrophy of the rest of the mucous membrane. That they grow usually from the middle turbinate, and especially from that part of it in the hiatus semilunaris, is due, probably, to the fact that there are some peculiarities in that portion of the nasal mucous membrane which lends itself to their development.

On examining the mucous membrane of a healthy nose it is found to be, both over the middle turbinate as well as the inferior turbinate and the septum, more or less the same. The only very appreciable difference is to be found in a certain amount of folding of the surface to be met with in the mucous membrane covering the middle turbinate, especially that part of it towards the hiatus semilunaris, while that over the inferior turbinate and septum is smooth. That these irregularities of the mucous membrane are more favourable to the development of polypi is likely. Gravity has probably also a certain influence in their production, as has also the suction action of the air drawn through the nose, somewhat forcibly in those cases where there is some hindrance to free respiration through it, as is likely to take place when the turbinates are swollen from catarrh, or hypertrophied, causing respiration to be almost entirely through the middle meatus. It would also be favoured by the superadding of some deflection of the nasal septum, &c., especially where there is interference with breathing through the inferior meatus with more patency of the middle.

While the commonest seat of origin of nasal polypi is from the mucous membrane covering the middle turbinate, that they are comparable to similar conditions of the septum and inferior turbinate must be admitted. Several such cases have been observed in which there were polypi growing from the septum or inferior turbinate, while the rest of the nasal mucous membrane was free from such formation, and which in no way differed from those ordinarily found growing from the middle turbinate except in that they were never pedunculated. That they are due to carious or necrosed bone I do not believe, as in all the cases I have examined I have met with none apart from syphilis in which the bone had undergone true necrosis. Even in those cases in which there is polypoid mucous membrane outside and inside some of the ethnoidal cells there is nothing in the intervening bone to suggest caries or necrosis. There is no marked infiltration of the tissues with round cells, and the bone while at one part showing large absorption spaces, at other places is thickened. In fact, this new formation and thickening of the bone at the base of the polypi is much more constant than degeneration. The same changes can also be fairly well presumed to occur in the mucous membrane lining the sinuses. This in no way differs anatomically from, and is simply a continuation of that in the nose. In fact, in suppuration of the sinuses they are always to be found lined with polypoid mucous membrane, and frequently with true polypi in them. The only difference lies in a, perhaps, greater abundance of round cells and glandular tissue, although the tissue still presents the characters of the mucous membrane with mucus infiltration.

(*To be continued.*)

